# A Rare Case of Disseminated Tuberculosis Presenting As a Frontal Headache and Photophobia in the UK

**DOI:** 10.7759/cureus.75986

**Published:** 2024-12-18

**Authors:** Faryal Khan, Robert Molloy, Umar-Khetaab Hanif, Muhammad J Hashmi, Rohan A Ahmed

**Affiliations:** 1 General Medicine, Midland Metropolitan University Hospital, Birmingham, GBR; 2 Radiology, Midland Metropolitan University Hospital, Birmingham, GBR

**Keywords:** active pulmonary tuberculosis, computed tomography, disseminated tuberculosis, intracranial tuberculous, psoas abscess

## Abstract

Tuberculosis is a disease caused by *Mycobacterium tuberculosis* (TB), demonstrating a vast clinical spectrum that can potentially involve all systems of the body. We present the case of a female in her late 20s, with an employment background in healthcare. She recently moved to the UK from India. The patient presented to the emergency department with a frontal headache and photophobia. Initial CT head demonstrated asymmetrical right-sided grey-matter hypoattenuation. Subsequent imaging showed multiple rim-enhancing lesions throughout the cerebral and cerebellar neuroparenchyma, scattered miliary nodules in both lungs, confluent necrotic mediastinal lymphadenopathy, collections in the psoas muscles bilaterally with extension on the left side into the iliacus, erector spinae and gluteal region and destructive sacroiliac joint involvement. This case highlights the complexity and variability of the presentation of extrapulmonary TB, subsequent imaging findings and diagnosis.

## Introduction

Tuberculosis (TB) is a disease caused by *Mycobacterium tuberculosis*, demonstrating a vast clinical spectrum that can potentially involve all systems of the body. Spread between humans is via droplet inhalation. Dissemination to the central nervous system (CNS) occurs due to the transit of mycobacteria following the rupture of tubercles into the blood [1,2].

Of the approximately 4400 new notifications of TB each year in the UK, only around 3% involve the CNS [3]. Presentations of CNS TB are more common in patients with suppressed immunity [4]. It can manifest as meningitis, tuberculoma or rarely arachnoiditis [4,5].

## Case presentation

We present the case of a 29-year-old female with an employment background in healthcare as she worked as a nurse. She moved to the UK three years ago from India. There was no significant medical history, and the patient was not taking any regular medications. There was no immediate family contact with TB.

The patient presented to the emergency department with a four-day history of a frontal headache and photophobia. No seizures or vomiting were reported. Further exploration of her history revealed a three-week history of drenching night sweats and unintentional weight loss of 5 kilograms.

On examination, she was febrile and mildly confused. No focal neurology deficits were identified, and the rest of the clinical examination was unremarkable. The C-reactive protein (CRP) was raised at 76 mg/L (range: 0-5 mg/L). The other blood parameters (including white cell count) were within normal limits (Table 1).

**Table 1:** Patient's relevant blood results.

Blood test	Value	Normal Range
White blood cell count	6.50	4-11 x10^9/L
Neutrophil count	4.85	2-7.5 x10^9/L
C-reactive protein (CRP)	76	0-5 mg/L

**Figure 1:**
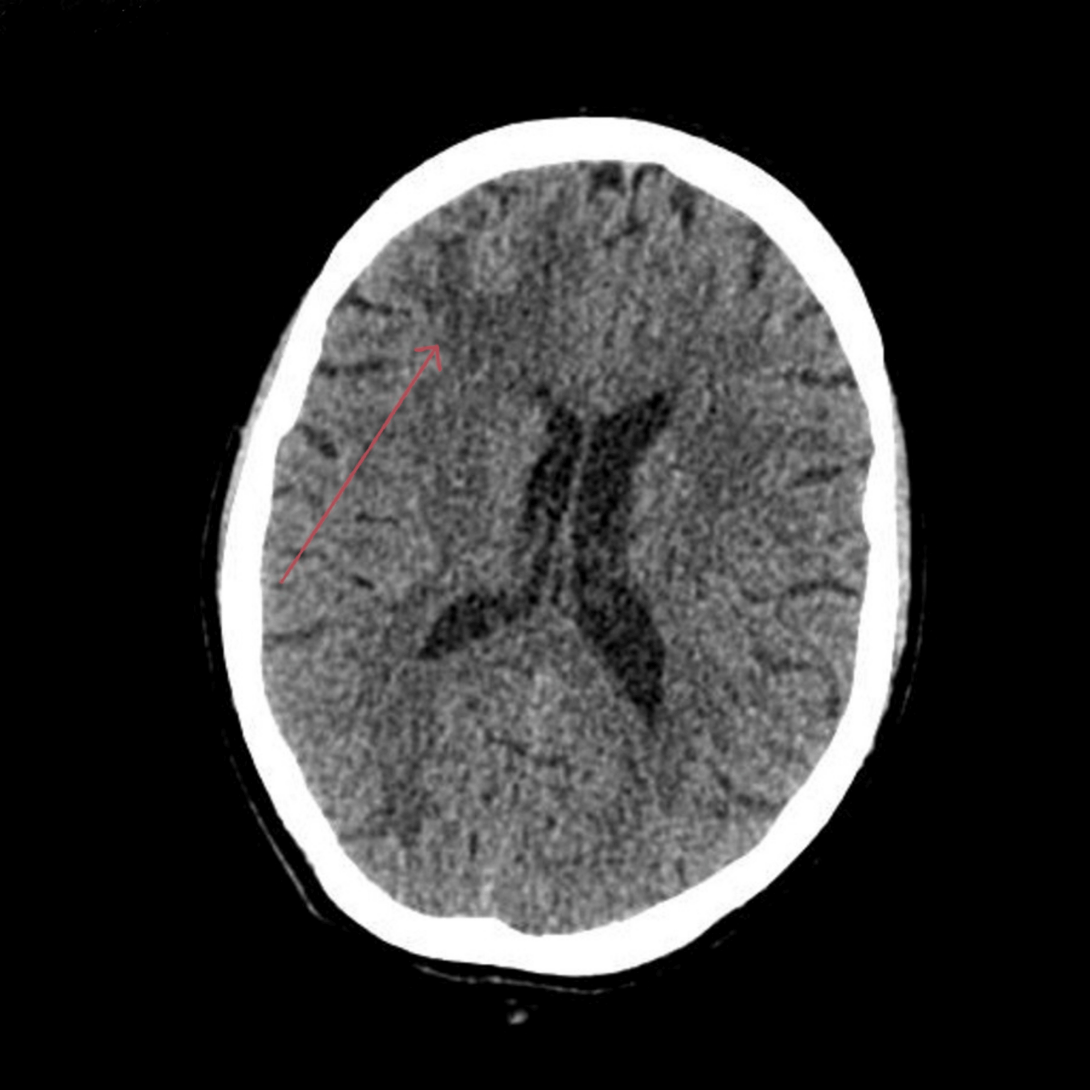
Non-contrast CT head showing asymmetrical right-sided grey-matter hypoattenuation, highlighted by a red arrow.

**Figure 2:**
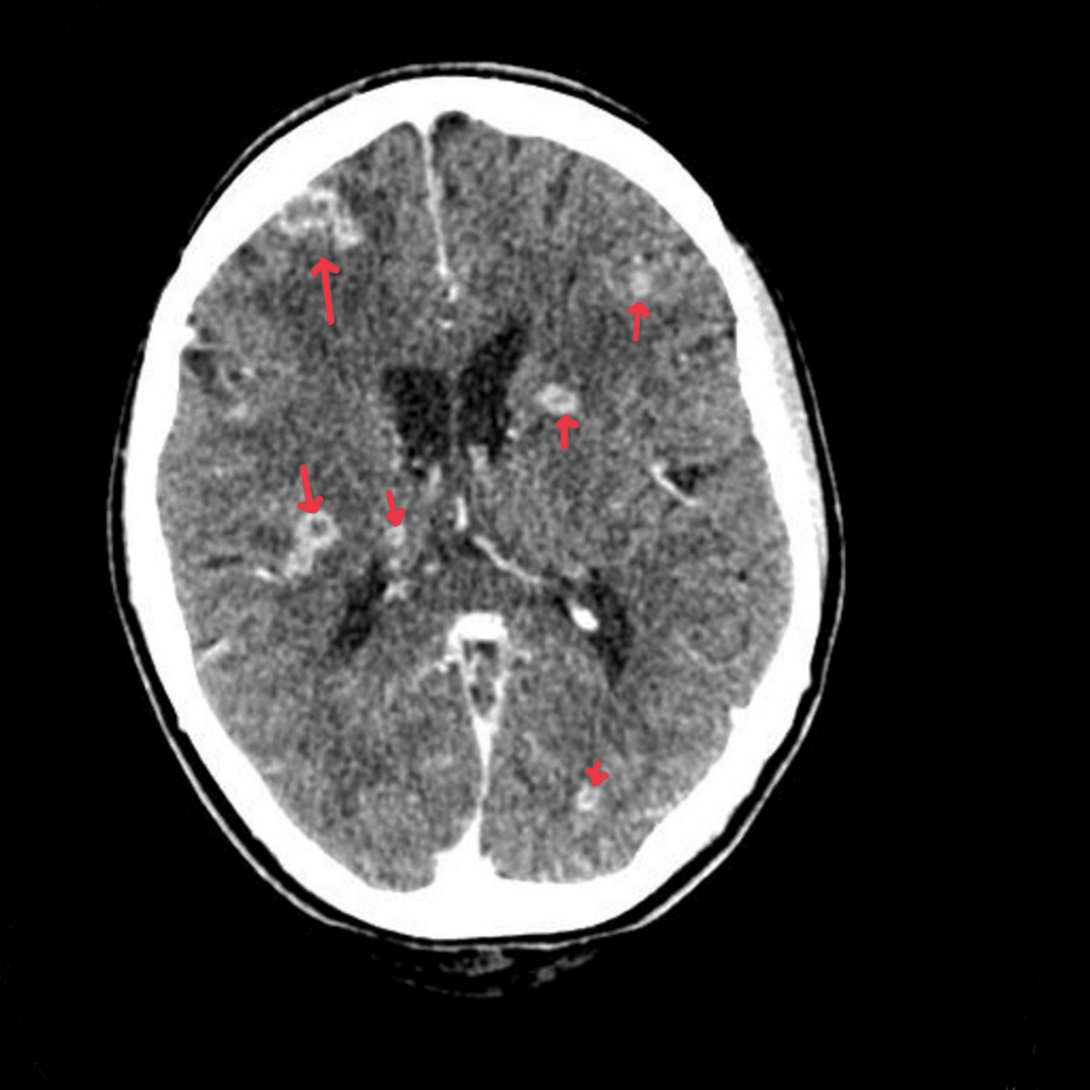
Contrast-enhanced CT head showing multiple ring-enhancing lesions.

**Figure 3:**
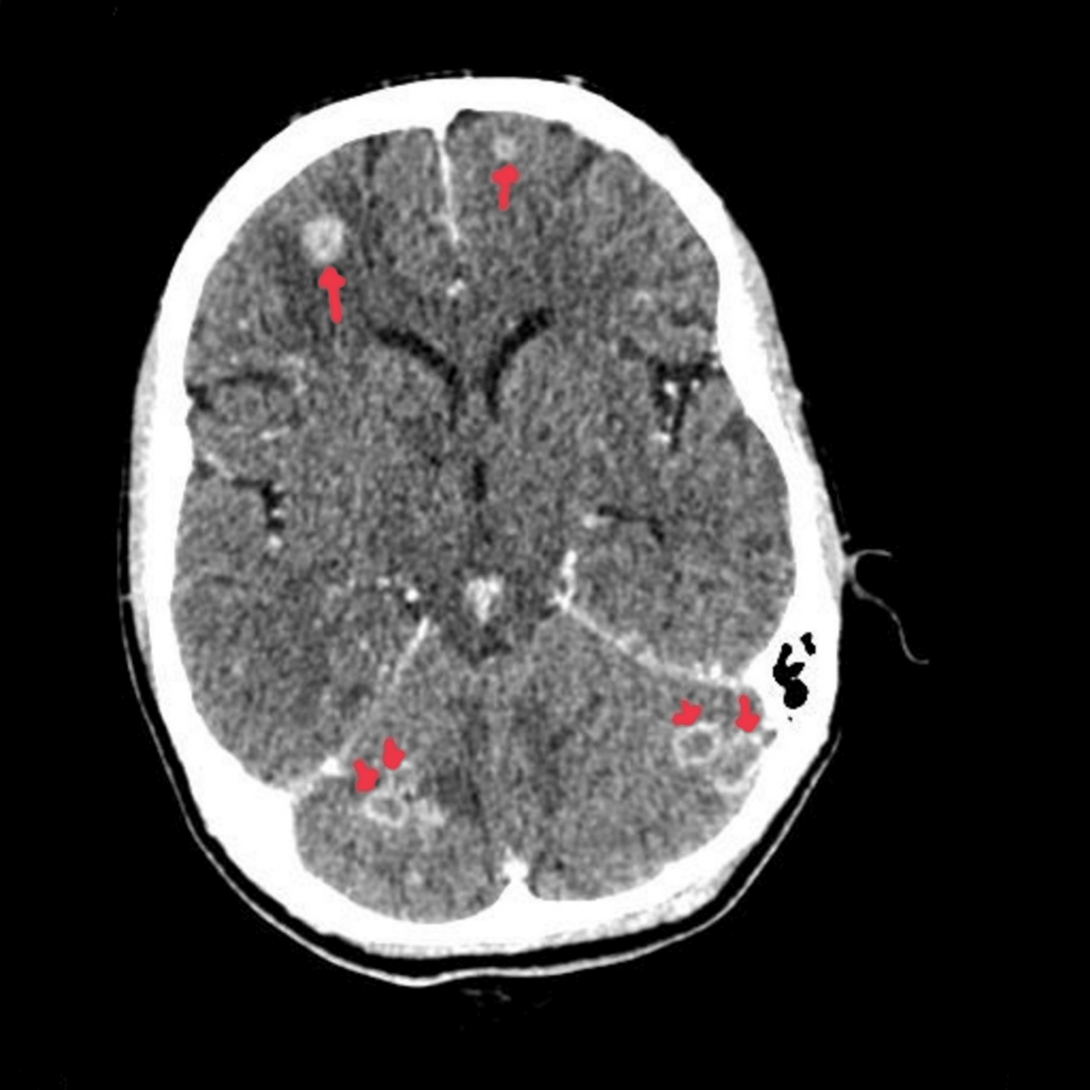
Contrast-enhanced CT head showing ring-enhancing lesions within the cerebellum.

In view of the new-onset confusion, the patient underwent a non-contrast-enhanced CT head. This demonstrated an ill-defined hypoattenuation located within the grey-matter junction of the right frontal lobe (Figure 1). This was followed by a contrast-enhanced CT head, which showed multiple rim-enhancing lesions throughout the cerebral and cerebellar neuroparenchyma (Figures 2-3). The patient was discussed with the local tertiary neurosurgical department, who initially felt that the most likely differential diagnosis was neurocysticercosis, but differentials including TB and malignancy would need to be considered.

**Figure 4:**
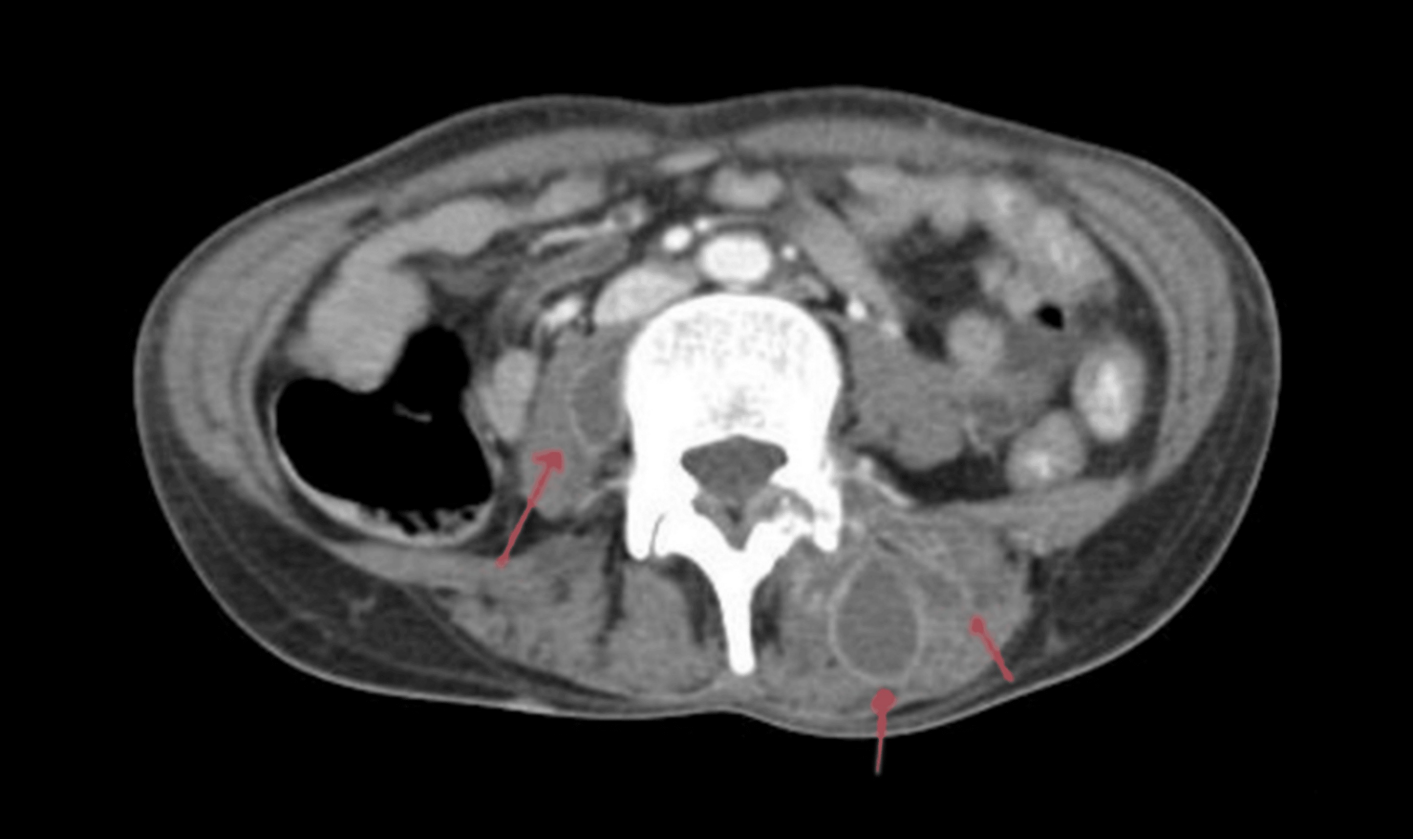
CT abdomen showing multiple abscesses within the right psoas muscle and left paraspinal muscles.

**Figure 5:**
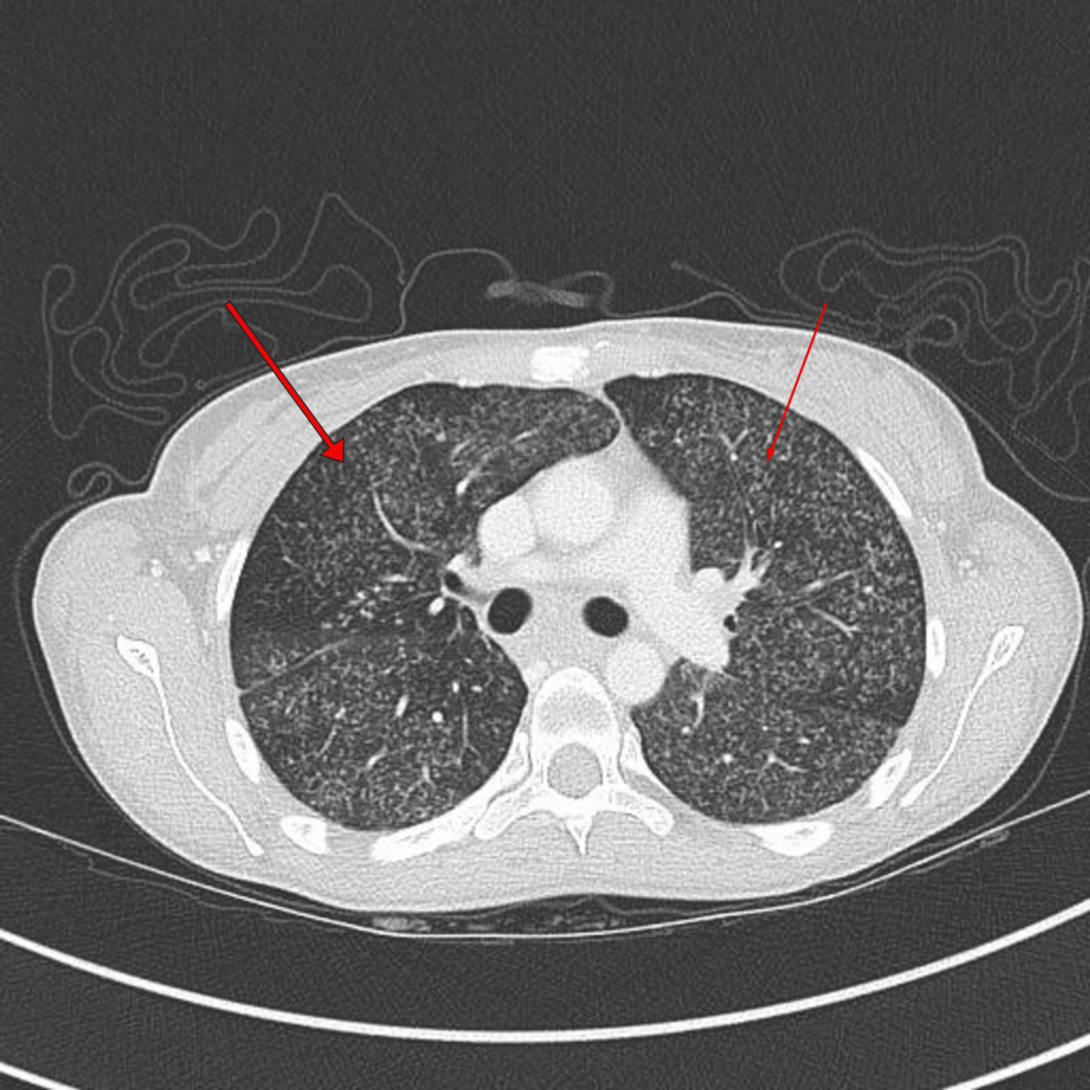
CT thorax showing hematogenously disseminated pulmonary tuberculosis, with arrows pointing to innumerable miliary deposits in both lungs.

A contrast-enhanced CT of the thorax, abdomen and pelvis was performed. This demonstrated innumerable scattered miliary nodules in both lungs, with confluent necrotic mediastinal lymphadenopathy. Large rim-enhancing collections were seen in the psoas muscles bilaterally, with an extension on the left side into the iliacus, erector spinae and gluteal region and destructive left sacroiliac joint involvement. Findings were consistent with acute disseminated TB (Figures 4-5).

Subsequent serology for neurocysticercosis and HIV was negative. Sputum was negative for acid-fast bacillus (AFB) on microscopy and negative for TB on culture at six weeks. An aspirated sample from the left psoas collection proved positive for AFB on microscopy, and TB was confirmed via PCR. The patient was commenced on oral anti-tuberculous treatment and has begun to make a progressive recovery. A follow-up culture, done two weeks later, confirmed very high confidence in mycobacterial identification.

**Figure 6:**
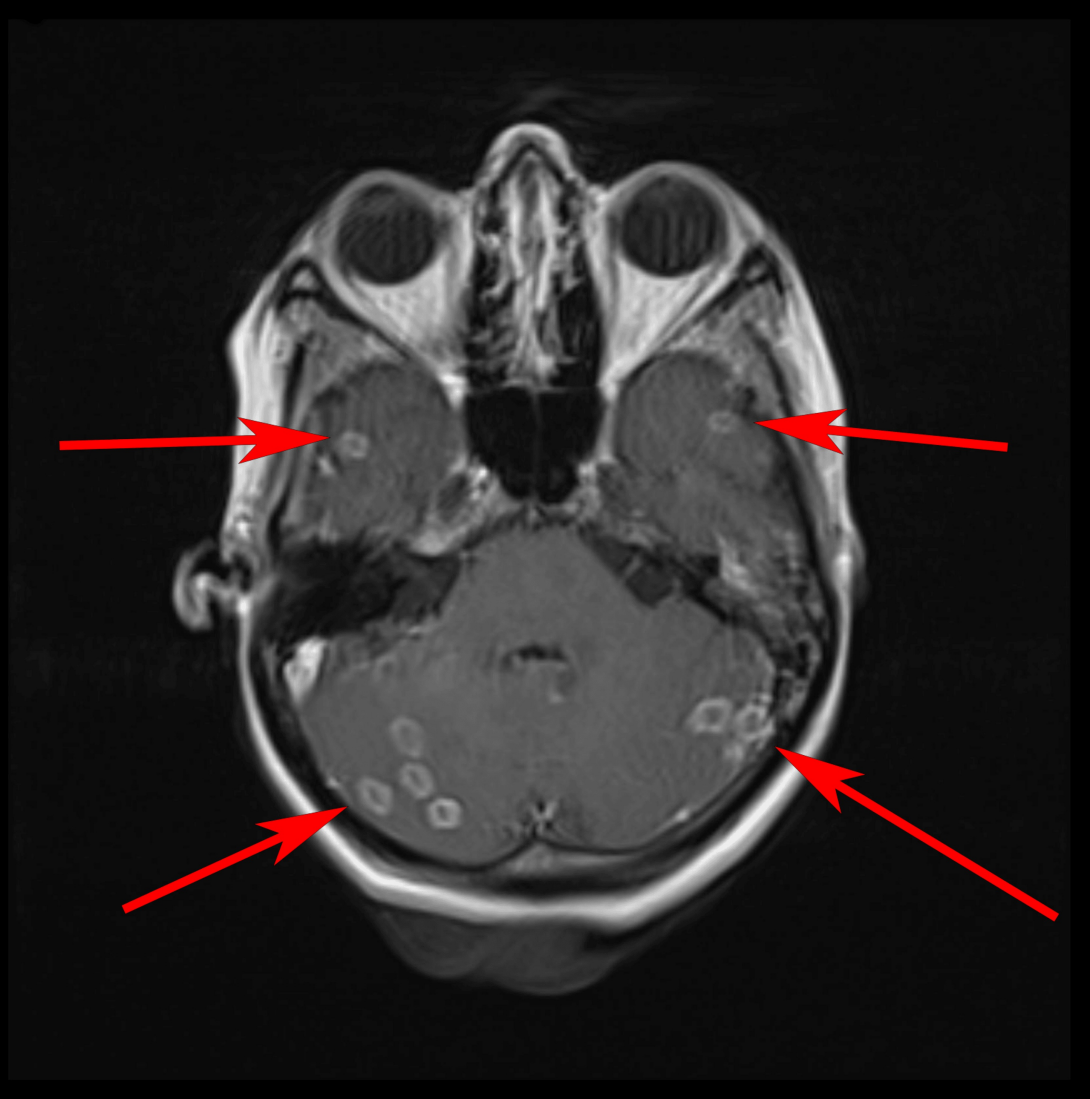
MRI head with arrows pointing to ring-enhancing lesions.

Pre- and post-contrast MRI of the brain demonstrated multiple innumerable marginally enhancing intracranial space-occupying lesions. The lesions were bright on T2-weighted and fluid-attenuated inversion recovery (FLAIR) sequences. There was surrounding vasogenic oedema that predominated in the right frontal and parietal locations. No restricted diffusion was seen (Figure 6).

## Discussion

In England, the incidence of *M. tuberculosis* infection has increased by 11% in 2023, which is the largest yearly increase since the year 2000, with the Midlands seeing a 5.95% increase. Up to 80% of active TB in England was reported from individuals born outside the UK, which has increased from 2022 to 7.2% in 2023 [6]. In addition to country of birth, factors such as overcrowded living conditions, homelessness, asylum seeker status and co-infection with HIV, all increase the likelihood of TB infection [7].

Although rare, the haematogenous dissemination of TB to the CNS can lead to the formation of intracranial lesions, such as tuberculomas, tubercular abscesses, and tubercular meningitis [5]. Tuberculomas are a specific type of granuloma, characterised by an irregular wall surrounding a necrotic mass that arises in response to chronic inflammation. The manifestations of CNS-TB can result in patients presenting with signs and symptoms of fever, malaise, headache, decreased consciousness, neck stiffness, photophobia, vomiting, increased intracranial pressure, focal neurological symptoms, and seizures [8-10].

Tuberculomas can be non-caseating granulomas, caseating granulomas with a solid centre, and caseating granulomas with liquid centres and each of these exhibits different radiological features [8,9]. Non-caseating granulomas on non-contrast CT show hypodensity to isodensity and on contrast CT show homogenous enhancement. On MRI non-caseating granulomas on T1-weighted images show low signal intensity and on T2-weighted images show high signal intensity. Caseating granulomas on CT and MRI contrast present with ring enhancement [8-12]. Other radiological findings associated with tuberculoma formation include perilesional oedema, microhemorrhages and predilection for the frontal or parietal regions [8-10,13]. It is important to note that tubercular abscesses appear larger and often as single lesions compared to tuberculoma [8,14].

Imaging of our patient revealed hypodensity in the right cerebral hemisphere on non-contrast CT, and T2-weighted MRI images demonstrated increased signal intensity. The patient also showed ring-enhancing lesions on contrast imaging. Ring-enhancing lesions are characterised as peripheral contrast enhancement surrounding central hypodensity or hypointensity, after the administration of contrast on CT and MRI, respectively [15]. This can be seen in conditions such as tuberculomas, neurocysticercosis, toxoplasmosis, and brain neoplasms [15,16].

Target signs are also a key radiological feature associated with tuberculomas. Target signs are where on CT contrast there is a ring of peripheral enhancement along with a central enhancement, which is usually due to calcification [10]. There is differing evidence on the specificity of ring enhancement and target signs for the diagnosis of tuberculoma. Stefanoni et al. and Bargalló et al. highlighted that target signs are also found in other diseases such as neurocysticercosis, brain metastasis, and lymphoma [17,18].

Furthermore, some lesions have a similar appearance and may be incorrectly identified as tuberculomas. For example, neurocysticercosis appears with a scolex, which is where the lesion displays point enhancement appearing as a "hole with a dot" [19,20]. Neurocysticercosis is a key differential when considering CNS-TB as demonstrated in our case report. *Taenia solium* (a helminth parasite) is endemic in Africa and Asia and causes neurocysticercosis due to the consumption of uncooked pork or the consumption of *T. solium* eggs [19]. It presents similarly to CNS-TB with seizures, headache, focal neurological symptoms and increased intracranial pressure [19].

Our case report and literature review have demonstrated the challenges in diagnosing tuberculomas, due to the varied and non-specific imaging features. As a result, alternative differential diagnoses such as neurocysticercosis were investigated. MRI can be useful in helping to differentiate between tuberculomas and neurocysticercosis. Further research and incorporation of the clinical application of advanced imaging modalities into routine clinical practice could enhance the accuracy of tuberculoma diagnosis.

The importance of early recognition of CNS-TB is increasingly relevant in the UK, due to the increasing incidence of TB. This highlights the importance of maintaining a high index of suspicion and recognition of the varied appearances of CNS-TB on imaging. Early consideration of TB in the differential diagnosis can facilitate timely investigations and interventions.

## Conclusions

In summary, we presented a rare case of disseminated TB in a young female presenting with a headache and fevers. TB can manifest in a variety of different ways and can affect multiple bodily systems. Diagnosis requires detailed history, radiology imaging, biochemical analysis and histopathology.

## References

[1] Burrill J, Williams CJ, Bain G, Conder G, Hine AL, Misra RR (2007). Tuberculosis: a radiologic review. Radiographics.

[2] Venter F, Heidari A, Galang K, Viehweg M (2018). An atypical presentation of tuberculomas in an immunocompetent host. J Investig Med High Impact Case Rep.

[3] (2024). TB incidence and epidemiology, England. https://www.gov.uk/government/publications/tuberculosis-in-england-2023-report-data-up-to-end-of-2022/tb-incidence-and-epidemiology-england-2022#:~:text=In%202022%20England%20remained%20a,WHO%20elimination%20target%20of%202035.

[4] Leonard JM (2017). Central nervous system tuberculosis. Microbiol Spectr.

[5] Saleh MA, Tu L, Mango M, Jacob M, Minja F (2022). Magic Dr.T? Tuberculous brain lesions in an immunocompetent patient-a case report. Radiol Case Rep.

[6] (2024). Tuberculosis incidence and epidemiology, England. https://www.gov.uk/government/publications/tuberculosis-in-england-2024-report/tuberculosis-incidence-and-epidemiology-england-2023.

[7] Litvinjenko S, Magwood O, Wu S, Wei X (2023). Burden of tuberculosis among vulnerable populations worldwide: an overview of systematic reviews. Lancet Infect Dis.

[8] Garg RK (1999). Tuberculosis of the central nervous system. Postgrad Med J.

[9] Bernaerts A, Vanhoenacker FM, Parizel PM (2003). Tuberculosis of the central nervous system: overview of neuroradiological findings. Eur Radiol.

[10] van Dyk A (1988). CT of intracranial tuberculomas with specific reference to the "target sign". Neuroradiology.

[11] du Plessis J, Andronikou S, Wieselthaler N, Theron S, George R, Mapukata A (2007). CT features of tuberculous intracranial abscesses in children. Pediatr Radiol.

[12] Zahrou F, Elallouchi Y, Ghannane H, Benali SA, Aniba K (2019). Diagnosis and management of intracranial tuberculomas: about 2 cases and a review of the literature. Pan Afr Med J.

[13] Roy S, Gupta SS, Muzaffar SN (2023). Intracranial microhemorrhages in a patient with tubercular meningitis (TBM): a case report. Cureus.

[14] Vidal JE, Cimerman S, da Silva PR, Sztajnbok J, Coelho JF, Lins DL (2003). Tuberculous brain abscess in a patient with AIDS: case report and literature review. Rev Inst Med Trop Sao Paulo.

[15] Chan KY, Siu JCW (2021). Magnetic resonance imaging features of cerebral ring-enhancing lesions with different aetiologies: a pictorial essay. Hong Kong J Radiol.

[16] Kuehnast M, Andronikou S, Hlabangana LT, Menezes CN (2020). Imaging of neurocysticercosis and the influence of the human immunodeficiency virus. Clin Radiol.

[17] Stefanoni G, Tironi M, Tremolizzo L (2014). Brain targets: can you believe your own eyes?. Neuroradiol J.

[18] Bargalló J, Berenguer J, García-Barrionuevo J, Ubeda B, Bargalló N, Cardenal C, Mercader JM (1996). The "target sign": is it a specific sign of CNS tuberculoma?. Neuroradiology.

[19] Del Brutto OH (2022). Human neurocysticercosis: an overview. Pathogens.

[20] Garg RK, Karak B, Sharma AM, Ojha R, Misra S (1999). Single CT (ring) lesion in epilepsy patients: a new observation. Indian J Pediatr.

